# KNeXT: a NetworkX-based topologically relevant KEGG parser

**DOI:** 10.3389/fgene.2024.1292394

**Published:** 2024-02-13

**Authors:** Everest Uriel Castaneda, Erich J. Baker

**Affiliations:** ^1^ Department of Biology, Baylor University, Waco, TX, United States; ^2^ School of Engineering and Computer Science, Baylor University, Waco, TX, United States; ^3^ Department of Mathematics and Computer Science, Belmont University, Nashville, TN, United States

**Keywords:** KEGG, KGML parser, python, NetworkX, KGML graph

## Abstract

Automating the recreation of gene and mixed gene-compound networks from Kyoto Encyclopedia of Genes and Genomes (KEGG) Markup Language (KGML) files is challenging because the data structure does not preserve the independent or loosely connected neighborhoods in which they were originally derived, referred to here as its topological environment. Identical accession numbers may overlap, causing neighborhoods to artificially collapse based on duplicated identifiers. This causes current parsers to create misleading or erroneous graphical representations when mixed gene networks are converted to gene-only networks. To overcome these challenges we created a python-based KEGG NetworkX Topological (KNeXT) parser that allows users to accurately recapitulate genetic networks and mixed networks from KGML map data. The software, archived as a python package index (PyPI) file to ensure broad application, is designed to ingest KGML files through built-in APIs and dynamically create high-fidelity topological representations. The utilization of NetworkX’s framework to generate tab-separated files additionally ensures that KNeXT results may be imported into other graph frameworks and maintain programmatic access to the original *x*-*y* axis positions to each node in the KEGG pathway. KNeXT is a well-described Python 3 package that allows users to rapidly download and aggregate specific KGML files and recreate KEGG pathways based on a range of user-defined settings. KNeXT is platform-independent, distinctive, and it is not written on top of other Python parsers. Furthermore, KNeXT enables users to parse entire local folders or single files through command line scripts and convert the output into NCBI or UniProt IDs. KNeXT provides an ability for researchers to generate pathway visualizations while persevering the original context of a KEGG pathway. Source code is freely available at https://github.com/everest-castaneda/knext.

## 1 Introduction

As network analyses become progressively feasible due to the increasing scalability and access to graph algorithms (Shannon et al., 2003; Lumsdaine et al., 2007; Hagberg et al., 2008) researchers are leveraging biological interaction graphs to derive new information, impute missing data, and drive decisions (Yue et al., 2019). Consequently, there often exists a need to visualize and extract information independent of a network’s original context and preserve the underlying graph structure to enable novel interrogation of the data (Bernstein et al., 2021).

Experimentally verified biological pathways play a key role in graphical analyses and are vital to hypothesis-testing and cross-validation experiments (Yu et al., 2017). One of the most widely used network pathway databases is the Kyoto Encyclopedia of Genes and Genomes (KEGG) (Kanehisa et al., 2022). KEGG hosts a series of experimentally verified and manually crafted biological pathways procured from various species and, most notably, all pathways can be externally downloaded as a network map written in KEGG Markup Language (KGML), a proprietary XML format. In order to parse, recreate, or convert KEGG pathways, KGML files require computational assistance for manipulation (Wrzodek et al., 2011). Currently, several software tools exist to parse KGML files: graphite, a bioconductor package which creates compound propagated genomic networks (Sales et al., 2012), CyKEGGParser, a Cytoscape application that fixes issues in KEGG-generated graphs (Nersisyan et al., 2014), KEGG2NET (Chanumolu et al., 2021), which converts KGML files into directed acyclic graphs, Biopython, a KGML parser for graphics rendering and data acquisition in Python (Cock et al., 2009), and KEGGParser, a MATLab-based parser and editor (Arakelyan and Nersisyan, 2013). Each parser fulfills a role in conforming, correcting, or converting pathways for use in other analyses, providing benefit where strict gene-only networks are required for input (Chen et al., 2017).

One fundamental challenge of creating automated KEGG parsers is to produce biologically-relevant representations of KGML pathways while maintaining the integrity of underlying data (Yu et al., 2017; Bernstein et al., 2021). Here we describe a python-based KEGG NetworkX Topological (KNeXT) parser that builds upon existing strategies by providing improved biologically-relevant representations of genetic networks and mixed networks from KGML data. To summarize, genetic networks are composed entirely of gene-gene interactions that avail themselves to semantic similarity analysis. (Díaz-Montaña et al., 2017). In contrast, mixed networks retain information regarding biological systems, such as interacting pathways, and chemical interactions Kanehisa et al. (2015). Due to the complex nature of KEGG graphs, parsers must produce output that can adapted for novel contexts, (Sales et al., 2012), and, furthermore, offer the ability to facilitate visualization, which is crucial for complex network data Sato et al. (2023). Hence, KNeXT converts KGML maps into tab-delimited files that are readily useable in other software analyses and visualization tools such as Cytoscape (Shannon et al., 2003) and NetworkX (Hagberg et al., 2008). An overarching difference between KNexT and other software is that it discriminates between genes and compounds in different topological configurations by utilizing the KGML file’s entry identifications as terminal modifiers. This allows users to derive networks in their original orientation and capture subgraphs. These topologies and overlapping nodes are a derivative of pathways which reflect spaciotemporally regulated complexes (Hurst et al., 2004) such as the cell cycle and cohesin loading (Litwin and Wysocki, 2018). KNexT features commands that enable users to recreate graphs as mixed networks or gene networks with compound propagation and “AND/OR group” parsing, similar to graphite (Sales et al., 2012). The resulting tab-delimited edge list also features notations of interactions that are derived from compound propagation or clique isolation and, if applicable, the original weights derived from the KGML file. KNeXT additionally provides access to a dictionary of nodes and their *x*-*y* axis coordinates for aiding in re-creation of the original KEGG layout in NetworkX.

## 2 Materials and methods

### 2.1 Overview

**FIGURE 1:**
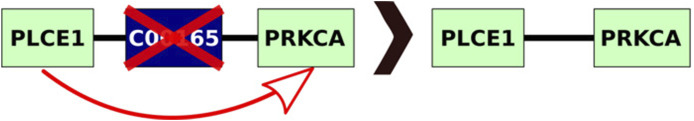
Example compound propagation. As described, KNeXT propagates genes by attaching edges between genes and then deleting the compound. KNeXT always retains unique identifiers in compounds, but for simplicity, the compound illustrated does not contain an entry identification number. KNeXT uses the shortest path algorithm in NetworkX to automate the propagation of compounds.

KNeXT is implemented in Python3 (v.3.9) and uses the NetworkX library (v.3.1) (Hagberg et al., 2008) for creating gene-only networks. Users may use the embedded KNeXT command, *get-kgml*, see Table 1 for a complete list of KNeXT commands, to retrieve species-specific KGML files using KEGG’s application program interface (API). Alternatively, users may specify local KGML files or directories as source materials. Pathway reconstruction is handled by the NetworkX shortest path framework. This approach implements compound propagation as described in (Sales et al., 2012) and Figure 1 to remove compounds from the network and avoid disconnected graph representations, and convert “AND/OR” type interactions into individual nodes (Sales et al., 2012). The shortest path framework is also used when transiting groups with undefined KEGG Orthology (KO) accession numbers.

**TABLE 1:** Summary statistics for all Kolmorogov-Smirnov tests for normality.

Graph	Variable measured	KS statistic	*p*-value
KNeXT	ppd	5.00 × 10^−1^	2.41 × 10^−73^
graphite	ppd	5.00 × 10^−1^	6.89 ×^−76^
KNeXT greedy	modularity	5.00 × 10^−1^	1.70 ×^−71^
graphite greedy	modularity	5.00 × 10^−1^	1.70 ×^−71^
KNeXT “Unique” greedy	modularity	5.10 × 10^−1^	3.13 ×^−75^
KNeXT Combo	modularity	5.00 × 10^−1^	1.70 × 10^−71^
graphite Combo	modularity	5.00 × 10^−1^	1.70 × 10^−71^
KNeXT “Unique” Combo	modularity	5.20 × 10^−1^	3.64 × 10^−157^

### 2.2 Parsing and output

**FIGURE 2:**
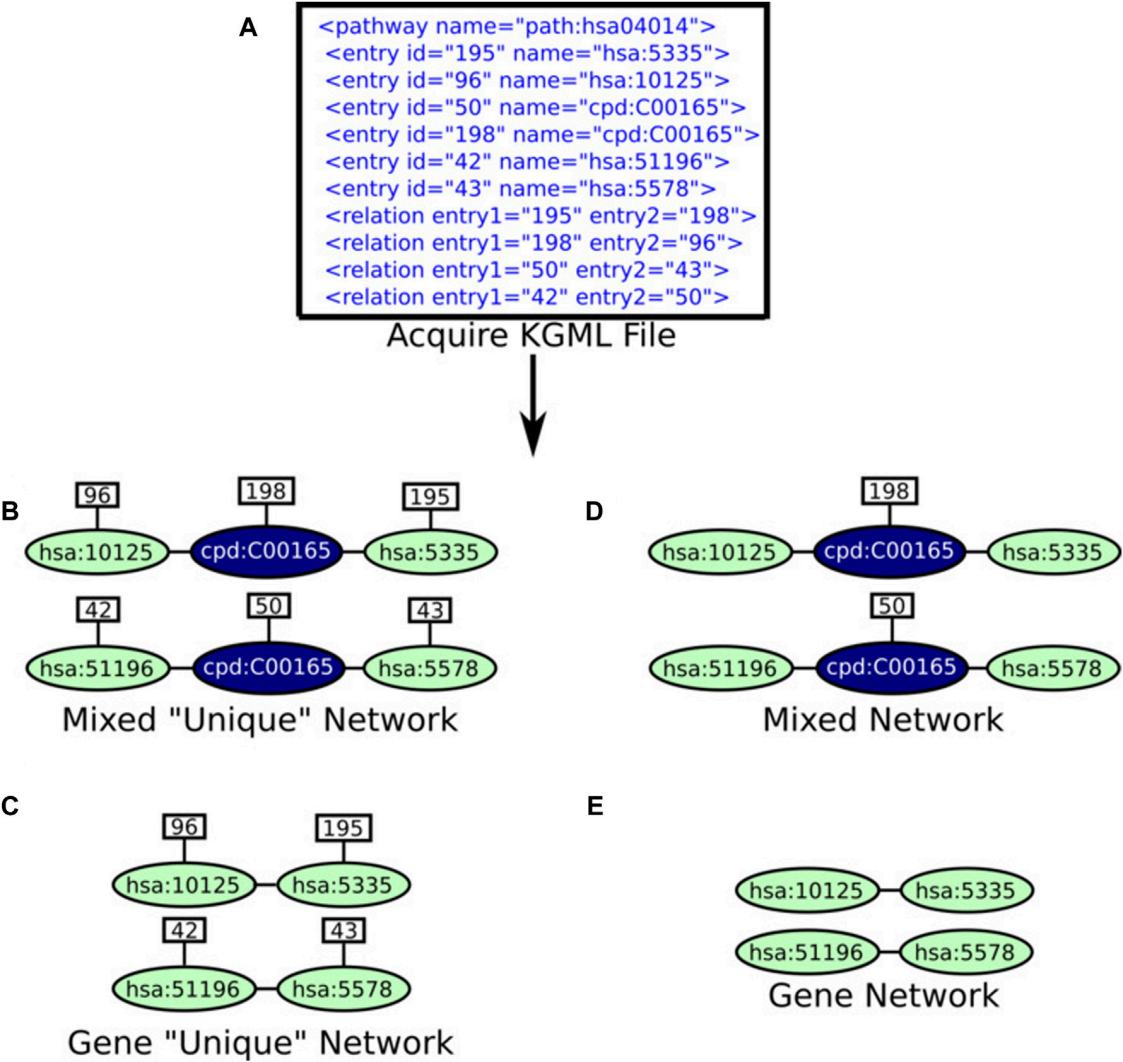
KNeXT’s schematic. **(A)**. KNeXT may acquire the KGML file or the user may use a downloaded KGML file as input. After, KNeXT parses the input then creates edges between genes, compounds, or other represented entities and attaches the entry identifier (id). There are four different outputs, which include the following: mixed, mixed unique, genes, and genes unique. **(B)**. The mixed “unique” network retains all entry ids as well as non-genomic entities such as compounds. **(C)**. The gene “unique” network retains only genes entities while also keeping entry ids to differentiate between genes involved in differing spatial topologies. **(D)**. The mixed network retains the entry ids for compounds to retain differentiation while removing all entry ids from gene entities. **(E)**. The gene network removes all entry ids, which is a similar output to graphite’s gene network.

KNeXT enables users to recreate KEGG pathways in the form of gene-gene networks or mixed networks consisting of a combination of genes, compounds, and pathways. See Figure 2 for an example of KNeXT’s process and Table 2 for programmatic descriptions.

**TABLE 2:** Commands and descriptions.

Primary command	Secondary command	Description
knext get-kgml	KEGG organism code	gathers all KGML files for the species specified
knext genes	[file]	parse a single KGML file as a gene-only network
	[folder]	parse a folder of KGML files as gene-only network
	–graphics	get TXT file of x-y coordinates of each node
	–unique	adds a terminal modifier to all nodes
	–names	uses an api call to acquire gene, compounds, and pathway names
knext mixed	[file]	parse a single KGML file as a mixed network
	[folder]	parse a folder of KGML files as a mixed network
	–graphics	get a TXT file of x-y coordinates of each node
	–unique	adds a terminal modifier to all nodes
	–names	uses an api call to acquire gene, compounds, and pathway names
knext convert	[file]	convert KNeXT output TSV file to UniProt or Entrez ids
	[folder]	convert KNeXT output TSVs in a folder to UniProt or Entrez ids
	–graphics [file]	convert a graphics file to UniProt or Entrez ids
	–graphics [path]	convert a folder of graphics files to UniProt or Entrez ids
	–unique	use only if your file(s) have terminal modifiers
	–uniprot	converts to UniProt ids (Entrez ids are default)

For gene only networks, KNeXT outputs a tab-separated value (TSV) file consisting of source and target gene with appropriate metadata, see Table 3 for example, Users may, in turn, visualize gene-gene output using NetworkX’s standard visualization libraries. Users wishing to maintain compounds, genes, and pathways may use the *mixed* command to output source and sink relationships, see Table 4 for an example. Lastly, using the*–names* command adds a “names” column for each accession, which enhances the ability to edit data, see Table 5.

**TABLE 3:** Example dataframe of gene-only network with metadata.

entry1	entry2	Type	Value	name
hsa:10000	hsa:1147	PPrel	–>	activation
hsa:100271927	hsa:22,800	PPrel	–>	activation
hsa:115727	hsa:4893	PPrel, PPrel	–>,–>	activation, activation
hsa:8503	hsa:7074	CPp	Custom	compound propagation
…	…	…	…	…

**TABLE 4:** Example dataframe of mixed networks with metadata.

entry1	entry2	Type	Value	name
cpd:C00035-92	hsa:22800	PCrel	—	binding/association
cpd:C00165-198	hsa:10125	PCrel, PCrel	–>,.>	activation, indirecteffect
hsa:11186	hsa:83593	PPrel	—	binding/association
…	…	…	…	…

**TABLE 5:** Example dataframe of mixed compounds and genes along with their and gene and compound names.

entry1	entry1_name	entry2	entry2_name
cpd:C00095-137	Levulose	hsa:5290	phosphatidylinositol-4,5-bisphosphate 3-kinase catalytic subunit alpha
hsa:10000	AKT serine/threonine kinase 3	hsa:6518	solute carrier family 2 member 5
hsa:207	AKT serine/threonine kinase 1	hsa:6518	solute carrier family 2 member 5
hsa:208	AKT serine/threonine kinase 2	hsa:6518	solute carrier family 2 member 5
…	…	…	…

For both gene-only and mixed-graphs, the*–unique* flag will generate a pathway with all genes and compounds with unique identifiers to avoid overlapping nodes. KNeXT does this by attaching unique terminal identifiers to each entry, ensuring that distal nodes are not bridged after compound propagation, see 2 B-C. Additionally, the terminal modifiers create distinct nodes that recapitulate correct positioning for pathway visualization. Users have the ability to use a*–graphics* flag to generate a dictionary of each node and its x-y coordinates, see Table 6 for an example. The resulting dictionary allows NetworkX to readily recapitulate KEGG style network visualization. All output can be readily converted into equivalent identifiers from other databases using the *convert* command. The conversion tool utilizes KEGG’s API to retrieve and map identifiers from Entrez (Sayers et al., 2022) and UniProtKB (The UniProt Consortium, 2023) IDs.

**TABLE 6:** Longform output of programmatic access to feature positions for input into NetworkX.

Node	Coordinates (x, y)
cpd:C01245-51	(889, 733)
cpd:C05981-22	(872, 282)
hsa:10000-23	(934, 283)
hsa:100137049-78	(1137, 533)
hsa:998-72	(1107, 445)
…	…

### 2.3 Data acquisition

Graphite version 1.48.0 was used for all pathway analysis. Since graphite contains its own database of pathways gathered from KEGG Sales et al. (2012), we captured 313 human pathways that are shared between both sources. See Supplementary Table S1 for a complete listing of all KEGG codes for each pathway used in this study and for R code used to export all pathways from the R environment. In addition, we created a ground truth graph using data from the UCSC Genome Browser using their filter to retrieve only database supported information (Kent et al., 2022). For simplicity, all relationships are represented as weightless undirected acyclic graphs.

### 2.4 Pathway analysis and statistical testing

We used two metrics for pathway analysis, per pathway difference (ppd) and modularity. For our analysis of ppd, we used the following: $ppd=\frac{set\left(A\right)-set\left(B\right)}{n}$ , where A is the set of all the edges in the query graph, KNeXT or graphite, B is the set of all the edges in the UCSC graph, and n is the number of pathways used. In order to determine significance, we conducted a Mann-Whitney-Wilcoxon test. Modularity was measured based on (Clauset et al., 2004a; Blondel et al., 2008) using the module in NetworkX (Hagberg et al., 2008). Briefly, $Q=\overset{n}{\underset{c=1}{\sum }}[\frac{L_{c}}{m}-\gamma {\left(\frac{kc}{2m}\right)}^{2}]$ where m is the number of edges in the graph, *L*_*c*_ is the number of intra-community links for community c, *k*_*c*_ is the sum of degrees in the nodes in community c, and *γ* is the resolution parameter. Here we used the default *γ* of one. For an equitable, non-biased approach, we included both graphite and KNeXT in the comparison of modularity against pathways generated using the*–unique* flag in KNeXT. Hence, we conducted a Kruskal Wallis (KW) Analysis of Variance (ANOVA) with a Dunnet’s (Dunn) *post hoc* cross comparison test. Dunn’s test was corrected for multiple testing using a Bonferroni correction. We used only non-parametric testing due to all measures being non-normal in distribution, see Table 1 for complete summary statistics of all Kolmogorov-Smirnov tests.

### 2.5 Community detection

One feature of KNeXT is its ability to parse pathways with terminal modifiers. Hence, we analyzed whether KNeXT’s “–unique” output graphs increase modularity using two different community detection algorithms. While the focus of this work is not to survey all applicable algorithms, we used greedy modularity (Clauset et al., 2004a), a module written in NetworkX (Hagberg et al., 2008), and pyCombo, a python wrapper around the C++ implementation of Combo (Sobolevsky et al., 2014). Both these algorithms have shown robust utility for capturing high modularity in human KEGG pathways (Rahiminejad et al., 2019). All algorithms were leveraged using default parameters, and, we used a seed of one for pyCombo. Briefly, the fast greedy community detection algorithm finds the community with the highest modularity from an iterative process that assess communities as community pairs are combined (Clauset et al., 2004a; b). pyCombo is a combination algorithm that finds communities with the highest modularity through using one of the following processes: combining communities, splitting communities, or moving nodes between communities (Sobolevsky et al., 2014).

## 3 Results

Automating the recreation of topologically relevant KEGG graphs from KGML files is difficult to due overlapping protein, gene, and compound identifiers. We do not know of any automated parsers that modify or interpret equivalent identifiers to create isolated connected components. KNeXT is intended to bridge this gap and our results provide a comparative analysis with a modern widely-used parser called graphite (Sales et al., 2012), which has been cited in several recent studies (Bianco et al., 2017; Gouy et al., 2017; Benedetti et al., 2020; Rahat et al., 2020; Hellstern et al., 2021; Liang et al., 2022).

**FIGURE 3:**
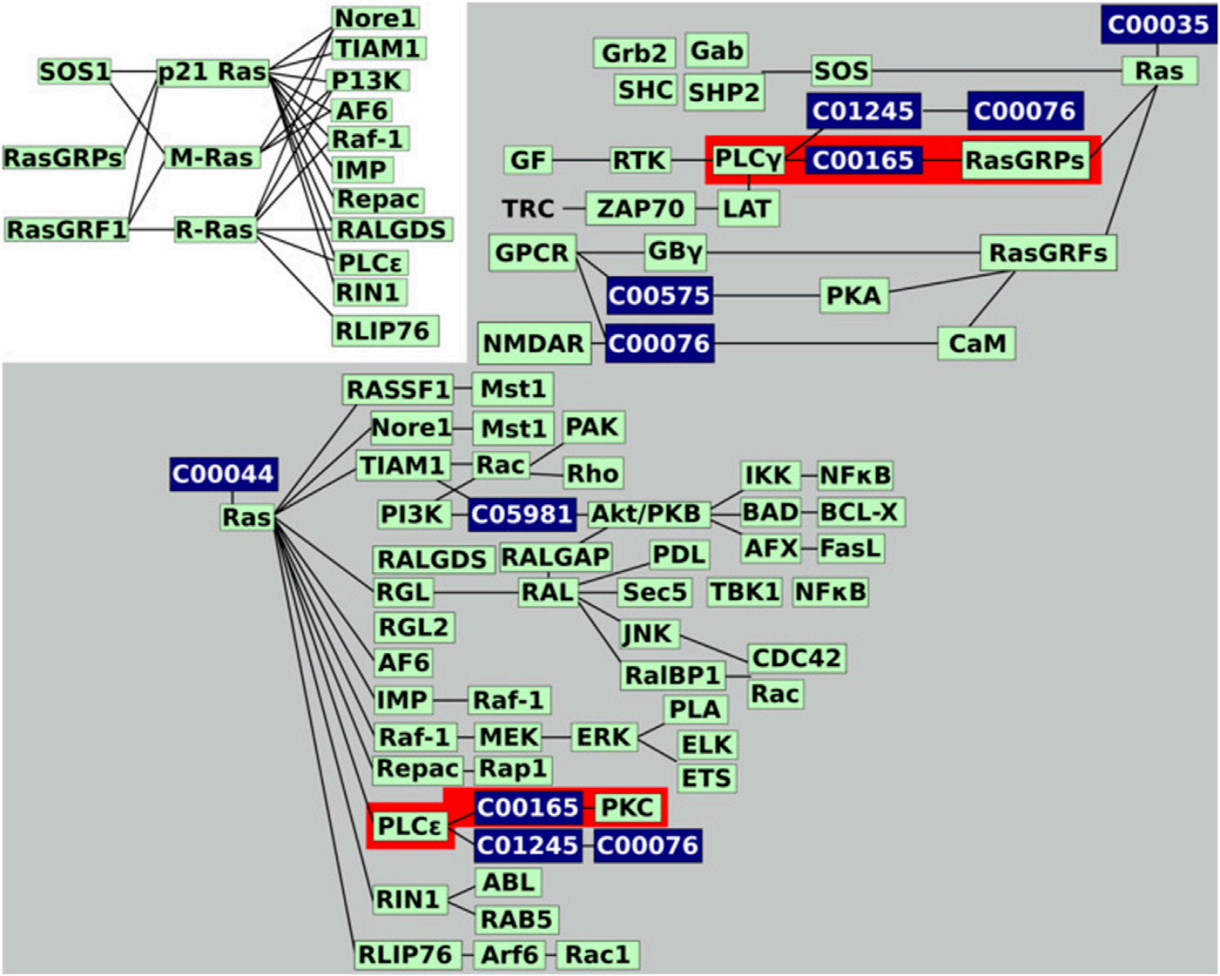
Recreated RAS signaling pathway. KEGG’s hsa04014 pathway image recreated. We reformatted the graph to undirected edges and emphasized the two connected components, in grey background, and the two neighborhoods, highlighted in red, we will be investigating. Genes are colored in green while compounds are in dark blue.

To illustrate the advantages of KNeXT we compare our novel approach to graphite using the *Homo sapiens* Rat Sarcoma (RAS) signaling pathway, a highly complex pathway with multiple effectors and features (Vojtek and Der, 1998). The KEGG pathway features two states, the active and inactive state, and a third independent state that outlines guanine nucleotide exchange factors and their effectors. Figure 3 is a recreation of the pathway as described in the KEGG website https://www.genome.jp/kegg-bin/show_pathway?hsa04014. To simplify the figure, we highlight pathways of interest and utilize undirected edges. Briefly, only the immediate neighborhood around compound C00165, DAG, is highlighted because it occurs in two subgraphs but forms connections with different genes based on which neighborhood it resides.

**FIGURE 4:**
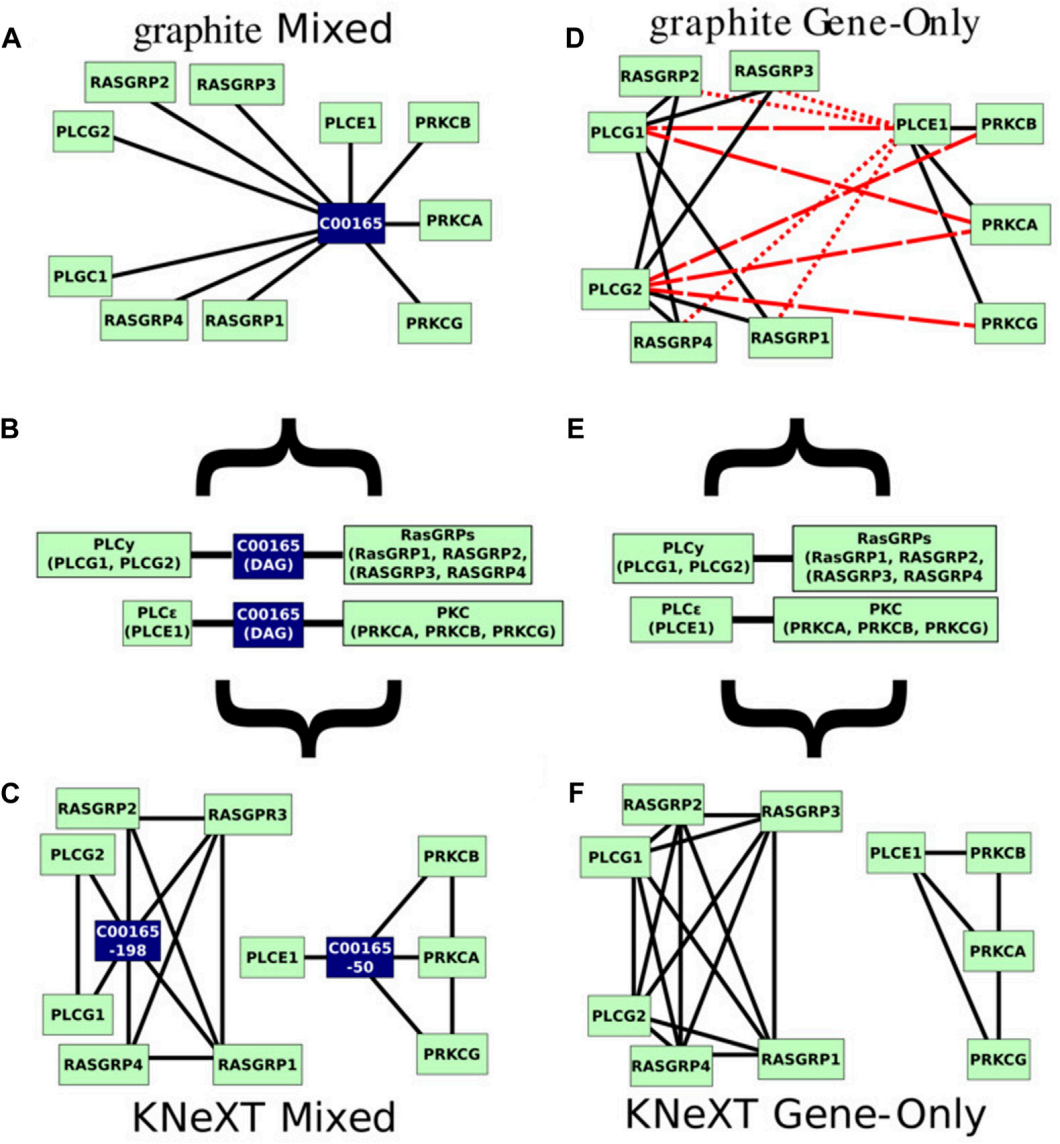
Mixed and gene only representations of the original KEGG pathway. **(A)**. The mixed, genes and compound, representation of the hsa04014 pathway generated by graphite using the *mixed* command. **(B)**. Pathway hsa04014s immediate neighborhood centered on compound C00165 (DAG). Type “AND” interactions are not individualized to emphasize what is originally given by the KGML files. Gene symbols within the type “AND” interactions are notated with parenthesis. **(C)**. The mixed genes and compound representation of the hsa04014 pathway parsed by KNeXT using the *parse-mixed* command. **(D)**. graphite’s gene only network representation of the hsa04014 pathway. Erroneous or otherwise misleading edges are marked in red dashes, and furthermore, edges which have no scientific evidence are marked in red dotted lines. **(E)**. Pathway hsa04014 from KEGG represented in gene only form. Type “AND” interactions are not individualized to emphasize the original KGML file format. **(F)**. The parsed hsa04014 pathway generated by KNeXT using the *parse-genes* command. In both cases, KNeXT illustrates higher accuracy when recapitulating the two distal neighborhoods.

Relying on unmodified KGML files, automated parsers are susceptible to overlapping nodes with the same identifiers, even when the nodes are in disconnected or distant neighborhoods (Figures 4A,D). As Figure 3 demonstrates, in the RAS pathway, DAG is represented in both the activate and inactive states. Although DAG is the exact same in either state, it exists in two distant neighborhoods or toplogies, see Figure 4B. As a result, when propagated with graphite, genes involved in PLC*γ* are bridged to both RasGRFs and PKC, creating a topology not reflected in the original KEGG diagram, compare Figures 4D,E. Figure 4D highlights edges in dashed red lines that do not support the original topology, and edges in dotted red lines are interactions not reflected in the University of Southern California Genome Browser Gene Interaction Track (Kent et al., 2022). While the latter is desirable, the former is misleading because it establishes connections between genes that are not indicated in the original KEGG pathway nor have any scientific evidence supporting its interactions (Kent et al., 2022). For example, the connection between genes PLCE1 and PLCG1 (Figure 4D) inferred by graphite does not appear in either KEGG topology, Figure 3, in contrast to the KNeXT result that retains neighborhood topology, Figure 4F. This is also illustrated when compounds are propagated without the KNeXT terminal modifier, Figure 4C. KNeXT is able to properly represent the relationship as two distinct connected components, Figure 4F.

**FIGURE 5:**
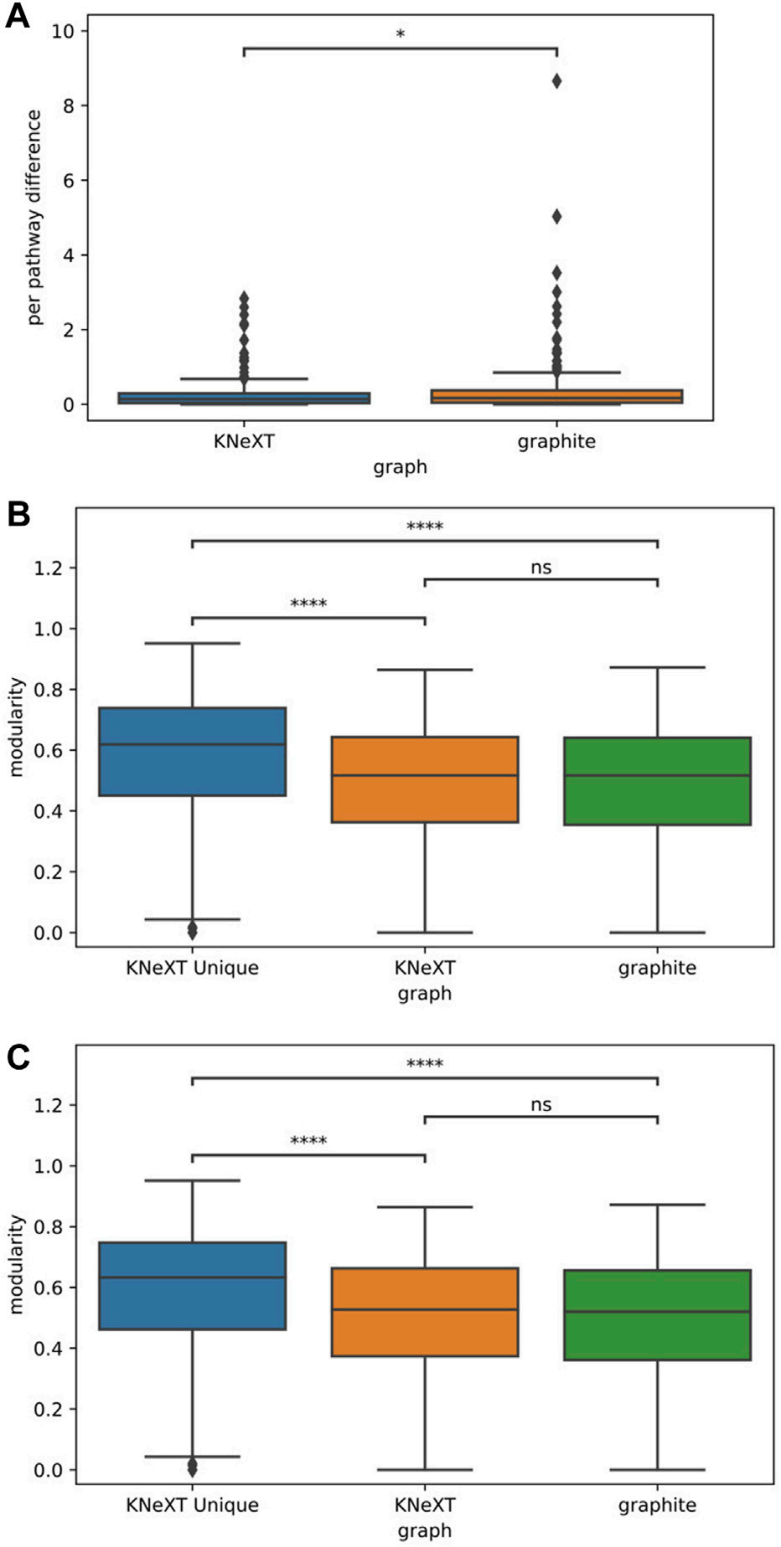
Results of pathway analyses. Probability value (*p*) annotation is as follows: ns: 0.050 < *p* <= 1.00,*: 0.010 < *p* ≤ 0.050, **: 0.0010 <p<= 0.01, ***: 0.00010 <p<= 0.0010, ****: *p* ≤ 0.0001 **(A)**. Results of a Mann-Whitney-Wilcoxon two-sided test. U-statistic is 4.66×10^4^ and *p* is 4.70 × 10^−2^. **(B)**. KW ANOVA results with Dunn’s *post hoc* test and a Bonferonni correction for a comparison of modularity using the greedy modularity algorithm for community detection. KW ANOVA results were significant *t* − *statistic* = 4.94×10^1^ and *p* = 1.90 × 10^−11^. While *post hoc* comparison showed non-significance between the graphite and KNeXT, *p* = 1.00, KNeXT “unique’s” average modularity was significantly higher than both KNeXT and graphite, *p* is 1.24 × 10^−9^ and 1.04 × 10^−8^, respectively. **(C)**. KW ANOVA results with Dunn’s *post hoc* test and a Bonferroni correction for a comparison of modularity using the Combo community detection algorithm. KW ANOVA results were significant *t* − *statistic* = 7.15×10^1^ and *p* = 3.06 × 10^−16^. While *post hoc* comparison showed non-significance between the graphite and KNeXT, *p* = 1.00, KNeXT “unique” was significantly higher compared to both KNeXT and graphite, *p* is 5.78 × 10^−12^ and 4.20 × 10^−11^, respectively.

We conducted a comparative analysis to extrapolate across all human pathways. Our first analysis was based on the validation between the edges constructed in a ground truth graph, which was gathered from the UCSC Genome Browser, and the edges generated in each respective parser, KNeXT and graphite. Figure 5A shows the results of a ppd between graphite and KNeXT. ppd was significantly higher for graphite, 0.045 *p*-value, compared to KNeXT indicating that a significant amount of unverified edges are inflated when compound propagation does not regard unique identifiers between each compound.

Our next analysis consisted of a survey of KNeXT’s ability to capture subgraphs using the*–unique* flag, which creates independent neighborhoods and unique topologies. This is the most prominent feature that differs from conventional parsers. We found that each pathway exhibited higher modularity compared to conventional parsing methods, see Figures 5B,C for results. This trend was consistent no matter which community detection algorithm we used, compare Figures 5B,C.

## 4 Discussion and conclusion

Topology, the spatial order of a graph’s edges and vertices, is a highly relevant aspect of network use in biology (Pavlopoulos et al., 2011; Gao et al., 2018). While identical gene or compound identifiers may be represented in disparate neighborhoods within the KEGG visualization framework, the existence of duplicate identifiers in the KGML file may prohibit recapitulation of original structure. Oftentimes, this will result in automated KGML parsers creating misleading or incorrect gene-gene or mixed-compound representations (Wrzodek et al., 2011; Arakelyan and Nersisyan, 2013; Nersisyan et al., 2014). Existing approaches, such as graphite, provide a tremendous utility to the bioinformatics and R communities and have demonstrated robustness across several additional databases (Sales et al., 2012). However, while KNeXT does not extend to additional databases, it does provide a novel automated KGML parser for the Python community while simultaneously addressing the pervasive issues driven by the KGML inclusion of duplicate identifiers. KNeXT is able to distinguish gene and compound localization within the larger topology without the need for post-processing modifications (Arakelyan and Nersisyan, 2013; Nersisyan et al., 2014). It also produces *x*-*y* axis localizations compatible with NetworkX, shown in Table 6, enabling rapid result visualization.

It is important to note that KNeXT achieves its ability to accurately reconstruct topology by adding unique modifiers to KEGG components via the*–unique* command. As a result, KNeXT outputs include modifier extensions to the original identifiers that need to be stripped before use in identifier mapping tools outside of KNeXT’s *convert* implementation. While KNeXT provides options for identifier mapping among Entrez (Sayers et al., 2022), UniProtKB (The UniProt Consortium, 2023), and KEGG (Kanehisa et al., 2022) ids, further experiments on fully realized, terminally-modified, KEGG pathways produced by KNeXT will be necessary to determine the impact of topology modification on downstream analysis. This may include the future development of tools for integrating KNeXT directly into larger packages such as *meta*Graphite (Sales et al., 2019), *netgsa* (Hellstern et al., 2021) or Cytoscape (Shannon et al., 2003). This will also include leveraging these terminally modified pathways in algorithms that require strict neighborhood embeddings such as graph autoencoders (GAE) (Lin et al., 2023). Since we have shown that these pathways exhibit a significant increase in modularity, it has been shown that GAEs may benefit from modularity-based prior communities when calculating embeddings (Salha-Galvan et al., 2022). Furthermore, the ability to swiftly isolate subgraphs will be useful to remove genes, which have a biased spatial pattern, such as housekeeping genes (Karathia et al., 2016).

In addition, we have noticed a lack of edges within UCSC’s graphical database. We attributed this to “OR” edges being unaccounted. Further research will be needed to investigate missing edges. Missing edges might be an artifact of automated parsers, and of course, our parser fills in these gaps without artificially inflating erroneous edges.

The KNeXT Python package will benefit users familiar with Python 3 and/or desire a command line interface to KGML downloading and parsing. KNeXT provides automated tools that perform analogous functions expected from modern KGML parsers (Sales et al., 2012) while preserving the vital overarching topological structure of KEGG source material. Our software reduces the complexity of isolating connected components, externally visualizing graphs, and reducing pathways into just their genetic components.

## Data Availability

The original contributions presented in the study are included in the article/Supplementary Material, further inquiries can be directed to the corresponding author.
