# Supplementary material for: KNeXT: a NetworkX-based topologically relevant KEGG parser
Source: Front Genet. 2024 Feb 13;15:1292394. doi: 10.3389/fgene.2024.1292394 (PMC10896898; doi:10.3389/fgene.2024.1292394)
Supplement: Supplementary file 1 [file DataSheet1.pdf]

Supplementary Material

Code we generated to export and gather all KEGG pathways from graphite. We parsed graphite pathways outside of the R environment in order to generate our per pathway difference metrics and conduct other analyses in Python 3.

```
kegg <- pathways("hsapiens", "kegg")
for (x in 1:length(names(kegg))) {
     names.list <- names(kegg)[x]
     code.name <- gsub(":", "", kegg[[x]]@id)
     p <- kegg[[names.list]]
     genes <- edges(p, which = "proteins")
     write.csv(genes, file = paste0("graphite_entrez/",
     code.name, ".csv"), row.names = FALSE)
```

Table 1 Kyoto Encylopedia of Genes and Genomes (KEGG) pathway code for all 314 human pathways used in analysis.Each of the KEGG codes in this table are pathways that were used that are present in both graphite's database and KEGG's website, `https://www.genome.jp/kegg/`.

hsa04014
hsa05211
hsa05033
hsa00030
hsa05214
hsa04911
hsa04151
hsa04940
hsa00970
hsa00071
hsa00565
hsa00430
hsa05110
hsa05144
hsa04310
hsa05216
hsa04670
hsa04630
hsa04613
hsa04971
hsa04713
hsa00920
hsa00260
hsa04623
hsa04216
hsa00220
hsa05034
hsa03015
hsa00740

hsa00983
hsa00130
hsa04612
hsa00591
hsa00340
hsa05418
hsa04915
hsa04650
hsa04215
hsa05150
hsa05203
hsa04927
hsa04148
hsa05204
hsa04625
hsa05169
hsa00830
hsa04923
hsa04270
hsa03440
hsa00785
hsa04722
hsa04211
hsa04672
hsa05330
hsa04916
hsa04658
hsa04920
hsa00500
hsa00563
hsa04978
hsa04144
hsa04973
hsa00760
hsa05412
hsa05133
hsa00230
hsa00860
hsa04550
hsa05208
hsa00280
hsa00900
hsa04066
hsa05146
hsa05215
hsa04926
hsa04917
hsa04918

hsa04392
hsa04964
hsa04122
hsa00480
hsa04530
hsa05222
hsa04614
hsa04974
hsa00400
hsa05220
hsa00410
hsa00910
hsa05022
hsa00062
hsa04924
hsa03250
hsa05134
hsa04912
hsa00650
hsa05206
hsa05016
hsa05205
hsa05014
hsa04340
hsa00250
hsa04950
hsa04060
hsa05217
hsa05231
hsa00524
hsa04659
hsa01524
hsa04115
hsa00562
hsa04110
hsa05132
hsa00380
hsa04931
hsa04068
hsa04724
hsa00780
hsa04140
hsa04611
hsa04217
hsa04080
hsa00440
hsa04012
hsa04913

hsa00592
hsa04720
hsa00630
hsa04742
hsa05221
hsa04371
hsa05160
hsa05010
hsa04062
hsa05031
hsa04726
hsa05210
hsa00290
hsa00240
hsa00330
hsa00052
hsa00604
hsa05235
hsa04022
hsa05165
hsa04657
hsa00310
hsa05145
hsa04136
hsa00982
hsa01523
hsa05143
hsa04660
hsa05414
hsa04390
hsa00603
hsa05410
hsa05416
hsa04218
hsa04024
hsa05135
hsa05320
hsa05012
hsa04061
hsa05140
hsa00470
hsa04936
hsa04620
hsa00053
hsa04010
hsa04020
hsa05200
hsa04728

hsa05223
hsa04979
hsa00561
hsa05321
hsa00061
hsa00190
hsa05417
hsa00730
hsa04921
hsa05131
hsa04910
hsa04610
hsa00515
hsa04510
hsa00531
hsa04721
hsa04370
hsa00600
hsa04213
hsa04145
hsa05230
hsa05170
hsa05202
hsa00450
hsa04928
hsa04960
hsa05166
hsa05332
hsa05207
hsa04730
hsa04727
hsa00232
hsa04934
hsa05030
hsa05225
hsa00520
hsa05168
hsa04071
hsa04064
hsa04972
hsa05100
hsa05017
hsa05032
hsa04961
hsa04725
hsa05212
hsa05142
hsa00513

hsa05120
hsa03018
hsa05171
hsa05322
hsa00140
hsa00120
hsa00020
hsa04070
hsa04130
hsa00051
hsa05164
hsa05310
hsa04919
hsa05213
hsa04622
hsa05218
hsa00270
hsa04621
hsa00670
hsa03460
hsa04141
hsa00590
hsa05162
hsa00360
hsa04150
hsa04520
hsa04380
hsa05226
hsa04137
hsa04925
hsa00040
hsa04976
hsa01521
hsa00790
hsa04072
hsa04930
hsa05415
hsa04015
hsa00620
hsa04261
hsa05323
hsa05219
hsa00532
hsa00512
hsa04668
hsa04114
hsa04750
hsa04210

hsa04744
hsa04740
hsa04360
hsa00350
hsa05130
hsa04350
hsa04540
hsa04723
hsa00640
hsa00980
hsa04152
hsa05224
hsa05167
hsa04512
hsa00100
hsa04933
hsa00534
hsa05163
hsa04962
hsa00770
hsa00510
hsa04922
hsa04260
hsa04664
hsa00750
hsa01522
hsa03320
hsa05161
hsa04975
hsa05020
hsa04970
hsa04666
hsa04514
hsa04662
hsa00601
hsa04932
hsa04810
hsa00564
hsa05152
hsa00010
hsa04330
hsa04935
hsa04914
hsa04714

Table 2 Graph metrics and per pathway difference for 10 KEGG graphs with high compound counts.

Pathway Codes	Pathway URL	Metrics	*graphite*	KNeXT
hsa:00140	`www.genome.jp/pathway/hsa00140`	number of edges	161	150
		number of nodes	29	29
		per pathway difference	0.51	0.48
hsa:00230	`www.genome.jp/pathway/hsa00230`	number of edges	992	930
		number of nodes	78	77
		per pathway difference	3.14	2.96
hsa:00980	`www.genome.jp/pathway/hsa00980`	number of edges	316	409
		number of nodes	44	44
		per pathway difference	1.00	1.30
hsa:00982	`www.genome.jp/pathway/hsa00982`	number of edges	224	222
		number of nodes	45	45
		per pathway difference	0.72	0.71
hsa:04080	`www.genome.jp/pathway/hsa04080`	number of edges	110	110
		number of nodes	108	108
		per pathway difference	0.14	0.14
hsa:00590	`www.genome.jp/pathway/hsa00590`	number of edges	168	162
		number of nodes	29	29
		per pathway difference	0.52	0.50
hsa:00350	`www.genome.jp/pathway/hsa00350`	number of edges	107	80
		number of nodes	24	24
		per pathway difference	0.33	0.25
hsa:04060	`www.genome.jp/pathway/hsa04060`	number of edges	246	232
		number of nodes	285	164
		per pathway difference	0.40	0.16
hsa:05020	`www.genome.jp/pathway/hsa05020`	number of edges	39	35
		number of nodes	20	20
		per pathway difference	0.12	0.11
hsa:00380	`www.genome.jp/pathway/hsa00380`	number of edges	57	54
		number of nodes	25	25
		per pathway difference	0.18	0.17
